# High energy implementation of coil-target scheme for guided re-acceleration of laser-driven protons

**DOI:** 10.1038/s41598-020-77997-w

**Published:** 2021-01-12

**Authors:** Hamad Ahmed, Prokopis Hadjisolomou, Kealan Naughton, Aaron Alejo, Stephanie Brauckmann, Giada Cantono, Simon Ferguson, Mirela Cerchez, Domenico Doria, James Green, Deborah Gwynne, Thomas Hodge, Deepak Kumar, Andrea Macchi, Rajendra Prasad, Oswald Willi, Marco Borghesi, Satyabrata Kar

**Affiliations:** 1 School of Mathematics and Physics, Queen’s University Belfast, Belfast, BT71NN UK; 2 Central Laser Facility, STFC Rutherford Appleton Laboratory, Didcot, OX11 0QX UK; 3 Institute of Physics of the ASCR, ELI-Beamlines Project, Na Slovance 2, 18221 Prague, Czech Republic; 4 Institute for Laser and Plasma Physics, University of Düsseldorf, Düsseldorf, Germany; 5 Dipartimento di Fisica Enrico Fermi, Università di Pisa, Pisa, Italy; 6 Extreme Light Infrastructure (ELI-NP) and Horia Hulubei National Institute for R & D in Physics and Nuclear Engineering (IFIN-HH), Reactorului No. 30, 077125 Magurele, Bucharest, Romania; 7 National Research Council, National Institute of Optics (CNR/INO), Research Unit “Adriano Gozzini”, Pisa, Italy

**Keywords:** Laser-produced plasmas, Plasma-based accelerators

## Abstract

Developing compact ion accelerators using intense lasers is a very active area of research, motivated by a strong applicative potential in science, industry and healthcare. However, proposed applications in medical therapy, as well as in nuclear and particle physics demand a strict control of ion energy, as well as of the angular and spectral distribution of ion beam, beyond the intrinsic limitations of the several acceleration mechanisms explored so far. Here we report on the production of highly collimated ($\sim 0.2^{\circ }$ half angle divergence), high-charge (10s of pC) and quasi-monoenergetic proton beams up to $\sim$ 50 MeV, using a recently developed method based on helical coil targetry. In this concept, ions accelerated from a laser-irradiated foil are post-accelerated and conditioned in a helical structure positioned at the rear of the foil. The pencil beam of protons was produced by guided post-acceleration at a rate of $\sim$ 2 GeV/m, without sacrificing the excellent beam emittance of the laser-driven proton beams. 3D particle tracing simulations indicate the possibility of sustaining high acceleration gradients over extended helical coil lengths, thus maximising the gain from such miniature accelerating modules.

## Introduction

Current interest in laser-driven ion accelerators, as a radically different, compact alternative to RF accelerators, stems from remarkable properties such as large particle flux, short pulse duration and exceptional beam emittance^1^. However, the large angular and spectral spread of the ion beams which are intrinsic to laser-driven acceleration mechanisms, pose significant technical challenges to their applicative use. For instance, application in cancer therapy would require the delivery of high energy protons (60–250 MeV)^2–4^ with narrow energy spread and sufficient particle flux at significant distances from the interaction targets, so that the extraneous radiation produced during the intense laser interaction can be shielded adequately. There has been recent significant progress in increasing the maximum proton energies delivered through laser-driven processes, with recent reports of the acceleration of near 100 MeV protons^5,6^, albeit with a broad spectral content and large angular spread, with an half cone divergence of $5^{\circ }-10^{\circ }$ at the highest energies.

Control of the beam divergence and of its energy spectrum have been key research objectives over the past decade, and a number of approaches using magnetic systems, target or plasma engineering^7–13^ have been explored for this purpose. The recently developed scheme based on helical coil (HC) targets^14^ offers, in this context, a miniature and versatile setup that, in addition to reducing the divergence and energy spread of the beams, has been shown to post-accelerate the guided protons at a rate of the order of GV/m. In this scheme, the electromagnetic (EM) pulse generated due to transient charging of an intense-laser irradiated foil^14–18^ is directed to travel along the helical path defined by an HC. The characteristics of the EM pulse are governed by the generation of hot electrons and their dynamics during the laser interaction and hence depend on a number of laser and target parameters^17,18^. While traveling along the windings of the HC, the EM pulse generates a strong electric field pattern which travels along the coil with a speed depending on the coil radius and pitch. Through a suitable choice of these parameters, it can be made to match the speed at which 10s of MeV protons travel. Deploying the coil at the rear side of the laser irradiated foil, protons within a narrow energy range are allowed to co-propagate with the travelling field pattern, enabling the synchronised proton bunch to be guided and post-accelerated simultaneously under the effect of the radial and longitudinal components of the electric field^14,19^.

In this article, we demonstrate the generation of highly directional beams (half-angle divergence $\sim 0.2^{\circ }$) of protons with energies up to $\sim$ 50 MeV and narrow energy spread ($\sim$ 10% FWHM), by employing HC targets at petawatt-class laser systems. The proton flux in the pencil beam at the spectral peak ($\sim$ 45 MeV) is of the order of $10^{12}$/MeV/sr, which is orders of magnitude higher than the fluxes typically generated by the TNSA mechanism at the high energy end of the spectrum^5,6,20,21^. Particle tracing simulations corroborate the experimental results, showing synchronous focusing and post-acceleration of transiting protons at a rate of $\sim$2 GeV/m, about four times larger than reported in the first demonstration of the HC technique^14^ and well beyond the capabilities of conventional RF accelerators. The scaling for Ti:Sa systems have been discussed in the ref.^14^, which shows that acceleration gradients of multi-GeV/m can be achieved with ultra-short, petawatt class lasers. A current limitation of the scheme will be discussed, together with a possible scheme to overcome it towards the production of beamlets at energies of therapeutic interest.

## Results

**Figure 1:**
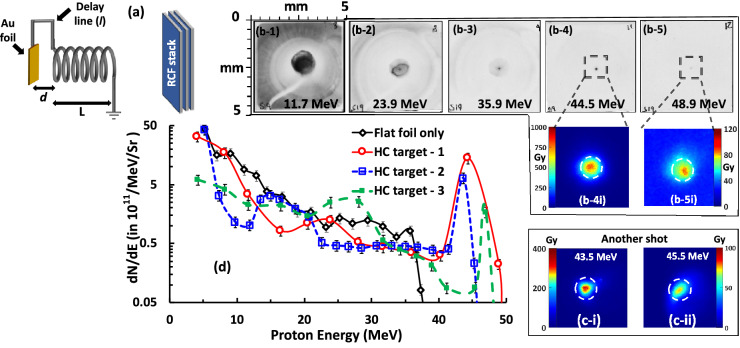
**(a)** Schematic of the setup (not to scale) used in the experiment. The HCs were made of 0.125 mm thick stainless steel wire and had pitch, internal diameter and length of $0.7 \pm 0.025 \, \hbox {mm}$, $0.7 \pm 0.015 \, \hbox {mm}$ and $7.7 \pm 0.1 \, \hbox {mm}$ respectively. The HC was placed at $1.5 \pm 0.05 \, \hbox {mm}$ from a $\sim \, 10 \, \mu \hbox {m}$ gold (Au) foil and connected to the foil by a delay line of length $6 \pm 0.1 \, \hbox {mm}$. The Radiochromic films (RCF) stack detector was placed at $60 \pm 1 \, \hbox {mm}$ from the interaction foil. Proton beam footprints captured by different RCF layers in the stack, corresponding to different proton energies, are shown in **(b-1)** - **(b-5)**. The scale shown at the top right corner of **(b-3)** refers to the RCF plane. Zoomed-in views of the dose profiles for the high energy pencil beams are shown in **(b-4i)**, **(b-5i)**, **(c-i)** and **(c-ii)**, where the last two snapshots are obtained from a second shot during the campaign using very similar laser and target parameters. The white-dashed circles on the zoomed-in views corresponds to the internal diameter of the HC. **(d)** shows the comparison between a typical proton spectrum obtained from reference flat foil targets and three HC target shots, RCF images from two of which (labelled as HC target-1 and HC target-2, respectively) are shown in **(b,c)**. The spectrum labelled as HC target-3 was obtained from a shot taken at similar interaction conditions, using a HC target syncing similar energy protons ($\sim$30 MeV) as in case of **(b,c)** with an internal diameter and pitch of $0.5 \pm 0.015 \, \hbox {mm}$ and $0.5 \pm 0.02 \, \hbox {mm}$ respectively. The on-axis proton spectra were obtained from the RCF data, as described in refs.^22,28^, considering the proton dose from the area enclosed by the black-dashed circles in the zoomed-in RCF views, corresponding to $0.33^{\circ }$ half angle divergence ($10^{-4} \, \hbox {sr}$). The error bars were estimated considering the error in dose conversion^22^ and uncertainties in background subtraction.

The data presented in this paper were collected from two experimental campaigns employing similar laser parameters, (see “Materials and methods” section for details). Fig. 1a shows a schematic of the HC target employed in the experiments, where the HC was placed at a few millimetres away from the interaction foil, and connected to the interaction foil by a metallic wire (the ‘*delay line*’). This configuration enabled precise control of the arrival time at the HC of the EM pulse relative to the arrival of the protons from the foil by either varying the length of the delay line, or the distance between the HC and the foil. Fig. 1b,c show results relating to the production of pencil beams of protons with a narrow energy bandwidth peaking at $\sim$ 45 MeV. As can be seen in Fig. 1b,c, the experimental data shows a highly collimated beamlet with energies up to $\sim$ 49 MeV, well beyond the maximum proton energies observed from reference flat foil shots taken during the campaign. The diameter of the central bright spot (containing more than 75% of the total flux) at the detector (Radiochromic film (RCF)) plane, 60 mm away from the target, is less than the internal diameter of the HC (shown by the black dashed circles in the zoomed-in views of the RCF images in Fig.1b,c). The spectral profile of the guided beam produced by the HC was reconstructed from the RCF data, as shown in Fig. 1d. Compared to the exponentially decaying spectra obtained from the reference flat foil shot, as typically expected from the TNSA mechanism, the on-axis proton spectra from the HC targets showed a pronounced, narrow spectral peak at $\sim$ 45 MeV with a full width at half maximum (FWHM) energy spread of less than 10% and peak prominence better than an order of magnitude. The number of protons at the spectral peak was of the order of $10^8/\hbox {MeV}$ (proton flux of the order of $10^{12}/\hbox {MeV/sr}$), which one could filter out (for instance, by using conventional accelerator optics^23,24^) from the rest of the spectrum to deliver narrow-band, collimated proton beams for applications.

**Figure 2:**
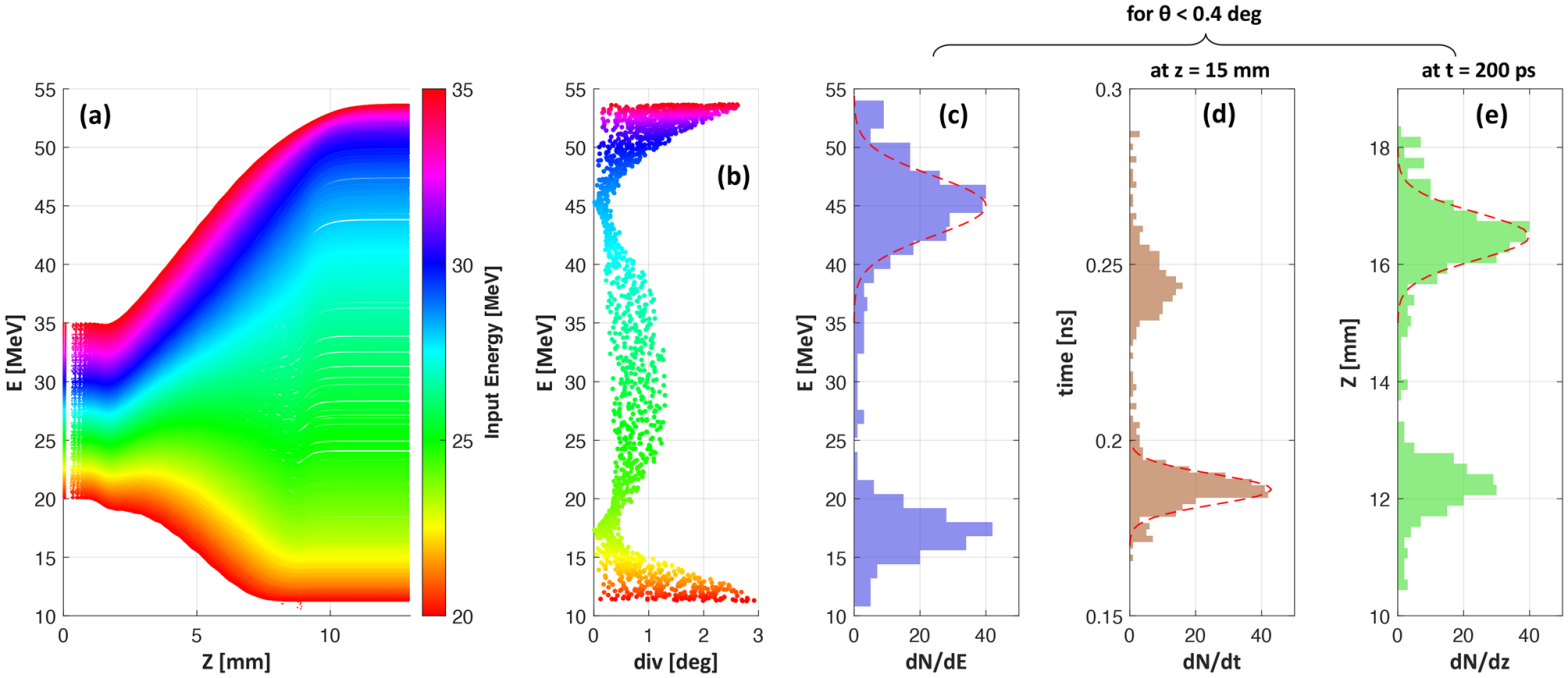
**(a)** and **(b)** highlight the dynamics of transiting protons through the HC obtained by particle tracing simulations, carried out using a source delivering a poly-energetic and divergent beam of protons in the setup shown in Fig 1a, with dimensions of the HC target used for the case shown in Fig. 1b. The simulation used the EM pulse profile measured in the experiment by proton probing, in a similar way as described in ref.^16^ and follows the propagation of protons with initial energy in the range of 25-35 MeV ( i.e. with energies close to synchronisation with the moving field for this specific target design). **(a)** shows the change in energy of protons while travelling along the HC axis *Z* (with proton source at *z*=0 mm and HC spanning from *z* = 1.5 to 9.2 mm, as per the experimental setup). **(b)** shows their divergence at $\textit{z} = 20 \, \hbox {mm}$, i.e. $\sim 1 \, \hbox {cm}$ after emerging from the HC. In both graphs, the vertical axis refers to final output energy of the protons while the colormap indicates their energy at the source. **(c–e)** show the energy spectrum, temporal and spatial spread respectively for protons in the collimated beam, having half angle divergence $\theta <0.4^{\circ }$.

The pencil beam of high energy protons results from the unique capability of chromatic guiding and post-acceleration offered by the HC targets. For a given HC radius and pitch, the strong focussing and accelerating fields move longitudinally along the HC axis with a fixed speed, which for the case shown in Fig. 1 was close to that of 30 MeV protons. At a given time the field pattern spans over a few windings of the HC as described in ref.^14^. While the maximum focusing field exists over the plane defined by the location the peak of the EM pulse, the accelerating field is optimum at a small distance (a few hundreds of microns depending on the HC radius and pitch) ahead of this position^14^. The delay line design of the HC targets, as used in the experiment, aids injecting the appropriate energy protons slightly ahead of the EM pulse peak, so that the protons clutch to the leading part of the field pattern and experience the optimum accelerating field.

Figure 2 illustrates the dynamics of transiting protons through the HC target, as reconstructed through particle tracing simulations employing the PTRACE code (see “Materials and methods” section). In Fig. 2a, the HC is seen accelerating efficiently the leading bunch of protons ($28 \pm 1 \, \hbox {MeV}$) entering the HC, whereas the lower energy protons entering later in time, i.e. after the arrival of the EM pulse peak, are decelerated due to the reversal of the longitudinal field, which points towards the proton source in the trailing part of the field pattern. Furthermore, as shown in Fig. 2b, within the accelerated bunch, the fastest protons which are sufficiently ahead of the EM pulse peak do not experience a strong focussing field and exit the HC without significant divergence reduction. Therefore, it is protons from a narrow slice of the input spectrum which emerge with an extremely low divergence and a significant energy gain—this simultaneous effect of energy-selection, focussing and post-acceleration is a capability unique to the HC targets. As indicated in Fig. 2, the HC target used for the shots shown in Fig. 1b acted efficiently on protons of $28 \pm 1 \, \hbox {MeV}$, delivering a pencil beam of $\sim$45 MeV with 6 MeV FWHM bandwidth (Fig. 2c), in a good agreement with the experimental results shown in Fig. 1d.

**Figure 3:**
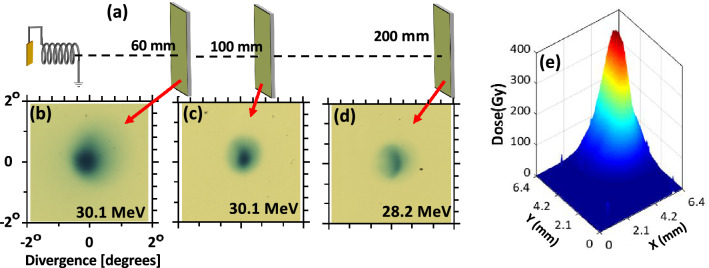
**(a)** Experimental setup (not to scale) used for characterizing the degree of beam collimation achieved by HC targets. Irradiating identical HC targets at similar laser conditions, proton beams were diagnosed by placing the RCF stack at different distances from the interaction foil. The HC targets used in this case were made of 0.125 mm thick Stainless steel wire of internal diameter, pitch and length of $0.7 \pm 0.015 \, \hbox {mm}$, $0.35 \pm 0.015 \, \hbox {mm}$ and $10 \pm 0.1 \, \hbox {mm}$ respectively , designed to synchronise optimally with $\sim$9 MeV protons and post-accelerate them to $\sim$30 MeV. **(b–d)** show spatial profiles of $\sim$30 MeV proton beams captured by the RCF stack placed at $60 \pm 1 \, \hbox {mm}$, $100 \pm 1\, \hbox {mm}$ and $200 \pm 1\, \hbox {mm}$ respectively. **(e)** shows the 3D dose profile of the proton beam shown in (c).

As shown in Fig. 1b,c, the diameter of the guided beam at 60 mm away from the target is smaller than the internal diameter (0.7 mm) of the HC, which indicates a half-angle divergence less than $0.33^{\circ }$. In order to characterise more precisely the beam divergence, beam profiles from three HC targets of $0.7 \pm 0.015 \, \hbox {mm}$ internal diameter and $0.35 \pm 0.015 \, \hbox {mm}$ pitch were recorded at different distances from the targets. Fig. 3b–d show beam profiles taken at 60 mm, 100 mm, and 200 mm from the interaction foil. As can be seen in Fig. 3e, the peak dose at 100 mm from the interaction foil is still several hundreds of Gy, providing ample scope for high dose delivery to a remote irradiation site, as required by numerous applications. The beam waist (FWHM) of $0.6 \pm 0.05 \, \hbox {mm}$ at 60 mm, expanded to $1.5 \pm 0.1 \, \hbox {mm}$ at 200 mm, corresponds to a nominal half-angle at half-maximum divergence of $\sim 0.2^{\circ }$. Taking the 0.7 mm diameter exit aperture of the HC as an upper estimate for the source size of the pencil beam, an upper limit for the beam’s normalised transverse emittance ($\simeq \beta _p r_0 \Delta \theta$, where $\beta _p$ is the ratio between proton velocity to speed of light in vacuum, $r_0$ being the source radius and $\Delta \theta$ being the half angle divergence of the beam) can be estimated as $0.15 \pi \, \hbox {mm mrad}$, indicating that the exceptional transverse emittance of TNSA beams (orders of magnitude lower than in RF accelerators)^25,26^ is substantially preserved.

The HC module is essentially a travelling-wave linear accelerator, where the accelerating field moves with the protons, so that the synchronised bunch of protons inside the HC experience a quasi-uniform field over an extended distance which favours the conservation of their longitudinal emittance. As can be seen in Fig. 2d,e, the collimated bunch of protons at around 45 MeV exits with an extremely low temporal and longitudinal spread (FWHM spreads of 10 ps and 0.94 mm respectively). Such ultra-short, localised bunch of ions could therefore be injected efficiently in subsequent stages of post-acceleration, for example using separate HC modules as proposed in ref.^14^.

## Discussion

**Figure 4:**
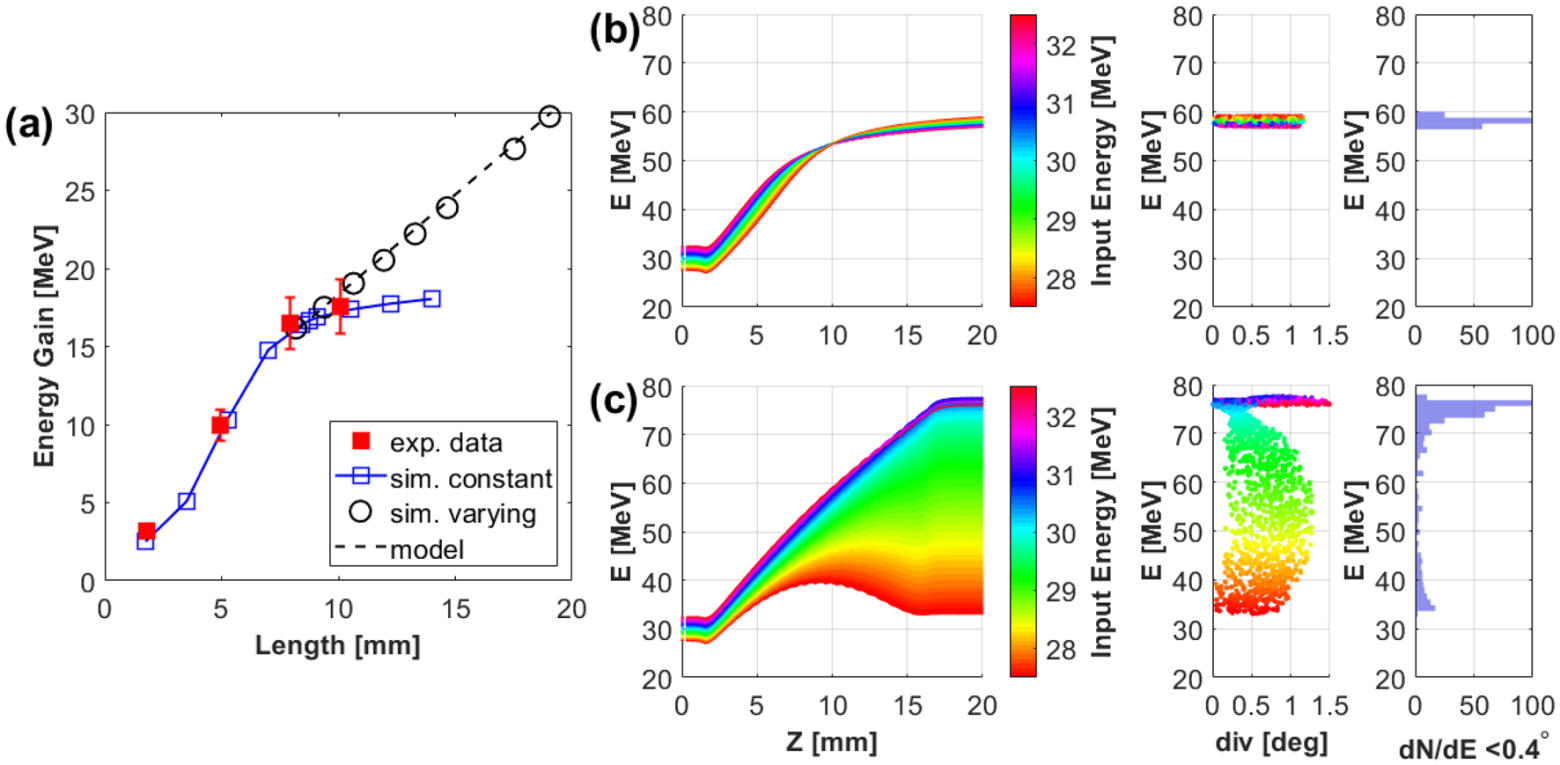
**(a)** Energy gain as a function of length of the HC target obtained from experiment (red) and simulations (Blue). The HC targets ( $0.7 \pm 0.015 \, \hbox {mm}$ internal diameter and $0.35 \pm 0.015 \, \hbox {mm}$ pitch) used in this case were similar to that used for the data shown in Fig. 3, except the length of HC which was varied from 2 to 10 mm. The simulations were carried out using the particle tracing code, as in Fig. 2, reconstructing the physical dimensions of the HCs used in the experiment and scanning for the HC length beyond the range studied in the experiment. The black circles show the simulated energy gain from a suitably designed varying pitch HC as calculated from the analytical model discussed in the text. **(b)** and **(c)** show dynamics of transiting protons (in a similar fashion as Fig. 2) through a constant and a variable pitched HCs, respectively. The HCs in both cases extend from *z*=1.5 to 16.5 mm with internal diameter 0.5 mm and initial pitch 0.5 mm. While the HC in **(b)** maintains a constant pitch over its full length, the pitch in (c) was varied according to Eq. (1), which gives a pitch increase of $\sim 13\mu \hbox {m}$ per turn. The beam divergence shown in **(b,c)** were taken at $z =100 \, \hbox {mm}$ and the energy spectra were plotted for a half-angle divergence $<0.4^{\circ }$.

While multi-staging of HC targets offers an attractive route towards a robust,‘all-optical’ accelerator, there is significant scope for performance optimisation of a single stage. A key parameter towards increasing energy gain is the length (L) of the accelerating module, which for the data shown in Fig. 1b was 7.7 mm. The effect of L on post-acceleration was studied experimentally by deploying HCs of different lengths (2-10 mm), while keeping the same radius and pitch (see Fig 4 caption for details). As can be seen in Fig. 4a, the experimental data shows a steady increase in energy of the guided protons with the HC length up to $\hbox {L} \sim 8 \, \hbox {mm}$. The particle tracing simulations indicate that a steady energy gain of $2.1 \pm 0.1 \, \hbox {MeV/mm}$ was maintained within this range. For longer HCs however, the net energy gain saturates, as can be seen clearly from the simulations, which are in agreement with the data obtained from the 10 mm long HC.

This saturation is due to dephasing between the accelerated bunch of protons and the travelling field pattern, since an increase in proton energy by tens of MeV will lead to protons overruning the field pattern, which for the case shown in Fig. 4a occurs after 8 mm of propagation. This problem can be mitigated by varying, continuously or in steps, either the radius or the pitch (or both), of the HC to maintain the travelling field pattern in phase with the protons.

The necessary modification to the HC geometry can be estimated by a simple analytical model as described here. Protons of energy $E_p(z) = \gamma _p(z)~m_{p}c^2=m_{p}c^2+T_{in} + G z$, where $m_p$ is the proton rest mass, *c* is the speed of light in vacuum, $T_{in}$ is the kinetic energy of the protons entering the HC and *G* is the energy gain per unit length inside the HC, need to remain at a fixed distance in front of the peak of the EM pulse, which requires the electric field pattern to travel with the same velocity as the protons at any given z. The velocity of the travelling field can be varied by changing either the radius or the pitch of the HC. For the purpose of this calculation we consider the pitch variation only as these are easier to implement in practice. The longitudinal velocity of the field pattern inside the HC can be expressed as $v_{z}(z)=c \beta _{_{EM}}~p(z)/\sqrt{(2\pi r)^2+p^2(z)}$, where *r* and *p* are the diameter and pitch of the HC respectively and $\beta _{_{EM}}=v_{_{EM}}/c$, where $v_{_{EM}}$ is the velocity of the EM pulse along a straight wire, which was measured experimentally as $\sim 0.98\pm 0.02 c$^14,16,17^. Equating the velocity of the travelling field to the proton velocity, one can find an expression for varying pitch to maintain a constant acceleration over an extended length,

1 $$\begin{aligned} p(z) = 2 \pi r \sqrt{\frac{\gamma _p^2(z)-1}{(\beta _{_{EM}}^2-1)\gamma _p^2(z)+1}} \end{aligned}$$

Figure 4a shows the predicted effectiveness of using varying pitch HCs over HCs fixed pitch HCs. While the accelerated protons start to dephase significantly after 8 mm of propagation inside the constant pitch HC, simulations shows that the energy gain can be maintained at the previous rate for an extended length of the HC by increasing the pitch according to the formula shown above. As we increase the acceleration gradient (*G*), either by increasing the amplitude of the EM pulse (as expected at multi-petawatt laser facilities^14^), and/or decreasing the radius (*r*) of the HC, using variable pitch HCs will be needed to maximize acceleration capabilities. As an example, Fig. 4b,c further elaborate the benefit of using a variable pitch HC over a constant pitch HC. For this comparison, HCs of 0.5 mm internal diameter were used which would provide $G \sim 3.0 \, \hbox {MeV/mm}$ with the same EM pulse produced in our experiment. While dephasing between proton bunch and electric field pattern in the constant pitch HC terminates the energy gain prematurely part way through the HC, the simulation suggests that a suitably designed variable pitch HC of 15 mm length could have produced a narrow bandwidth pencil beam of $\sim$75 MeV, i.e at an energy already adequate for treating ocular tumours or subcutaneous cancers for instance, and that could be further enhanced by deploying successive, separately driven and optimized HC stages.

## Materials and methods

### Experiment

Experiments were conducted at two different facilities, namely the Titan laser system at Lawrence Livermore National Laboratory (LLNL, USA) and the VULCAN Petawatt (VPW) system at Rutherford Appleton Laboratory (RAL, UK). They are both Nd:Glass based laser systems operating at central wavelength of $1.053 \, \upmu \hbox {m}$. In the Titan experiment, CPA pulses of duration $600 \pm 100\, \hbox {fs}$ and energy $150 \pm 25 \, \hbox {J}$ were focused on target by an f/3 off-axis parabola to a spot of $7 \pm 0.5 \, \upmu \hbox {m}$ FWHM delivering peak intensity $( 2 {\pm 1})\times 10^{20} \, {\hbox {W/cm}}^2$. In the second experiment, VPW delivered laser pulses of $1 \pm 0.1 \, \hbox {ps}$ duration with energy $300 \pm 50 \, \hbox {J}$. The laser pulses were focused by an f/3 off-axis parabola to a spot of $5.5 \pm 0.5 \, \upmu \hbox {m}$ FWHM, resulting in peak intensity $(3.5{\pm 1}) \times 10^{20} \, {\hbox {W/cm}}^2$. In both experiments, $10 \, \upmu \hbox {m}$ thick gold foils were used for proton generation, HCs were made of 0.125 mm stainless steel wire and the laser was incident at $20^{\circ }$ to the target normal. The spatial and spectral distribution of the proton beams was characterised by deploying a stack of dosimetrically calibrated Radiochromic films (RCF)^22^. The proton spectra were reconstructed by spectral deconvolution of the dose deposited in the RCF layers^27^, by using an iterative algorithm similar to the procedures used in refs.^22,28^. Starting from the last RCF layer in the stack, the final spectrum is produced by calculating spectra between Bragg peak energies of consecutive RCF layers, while considering the energy response of the RCF layers (simulated by SRIM^29^) in the stack and subtracting the dose contribution in a given layer by the protons stopping deeper in the stack.

### Simulations

The particle tracing simulations presented in this paper were performed using the PTRACE code^30^, which simulates the propagation in 3D of protons from source to detector through the region where e.m. fields are present in this case the field pattern produced by the EM pulse travelling along a HC target. The protons transit through the HC together with the co-propagating electric field associated to the travelling EM pulse. The protons are traced by computing relativistic equations of motion using a Runge-Kutta fourth-order algorithm coupled with an adaptive step size monitoring routine. The HC was modelled in PTRACE using a cylindrical co-ordinate system and the physical dimensions as used in the experiment. An EM pulse of peak linear charge density $50 \, \upmu \hbox {C/m}$, 5 ps half-maximum rise and 15 ps half-maximum decay, similar to that measured experimentally in both the campaigns using the technique of self-probing (described in the ref^16^), was set to travel along the HC wire. In the delay line configuration, the proton source was modelled as a point source located on the axis of the HC at a given distance from the entrance plane of the HC, emitting protons towards the HC with a given energy spectrum and divergence, mimicking the proton beam produced by the reference flat foil target.
